# Digital Approaches for a Reliable Early Diagnosis of Psoriatic Arthritis

**DOI:** 10.3389/fmed.2021.718922

**Published:** 2021-08-11

**Authors:** Filippo Fagni, Johannes Knitza, Martin Krusche, Arnd Kleyer, Koray Tascilar, David Simon

**Affiliations:** ^1^Department of Internal Medicine 3 - Rheumatology and Immunology, Friedrich-Alexander University (FAU) Erlangen-Nürnberg and Universitätsklinikum Erlangen, Erlangen, Germany; ^2^Deutsches Zentrum fuer Immuntherapie, FAU Erlangen-Nuremberg and Universitätsklinikum Erlangen, Erlangen, Germany; ^3^Department of Rheumatology and Clinical Immunology, Charité - Universitätsmedizin, Berlin, Germany

**Keywords:** psoriatic arthritis, telemedicine, eHealth, mHealth, digital rheumatology, psoriasis

## Abstract

Psoriatic arthritis (PsA) is a chronic inflammatory disease that develops in up to 30% of patients with psoriasis. In the vast majority of cases, cutaneous symptoms precede musculoskeletal complaints. Progression from psoriasis to PsA is characterized by subclinical synovio-entheseal inflammation and often non-specific musculoskeletal symptoms that are frequently unreported or overlooked. With the development of increasingly effective therapies and a broad drug armamentarium, prevention of arthritis development through careful clinical monitoring has become priority. Identifying high-risk psoriasis patients before PsA onset would ensure early diagnosis, increased treatment efficacy, and ultimately better outcomes; ideally, PsA development could even be averted. However, the current model of care for PsA offers only limited possibilities of early intervention. This is attributable to the large pool of patients to be monitored and the limited resources of the health care system in comparison. The use of digital technologies for health (eHealth) could help close this gap in care by enabling faster, more targeted and more streamlined access to rheumatological care for patients with psoriasis. eHealth solutions particularly include telemedicine, mobile technologies, and symptom checkers. Telemedicine enables rheumatological visits and consultations at a distance while mobile technologies can improve monitoring by allowing patients to self-report symptoms and disease-related parameters continuously. Symptom checkers have the potential to direct patients to medical attention at an earlier point of their disease and therefore minimizing diagnostic delay. Overall, these interventions could lead to earlier diagnoses of arthritis, improved monitoring, and better disease control while simultaneously increasing the capacity of referral centers.

## Introduction

Psoriasis (PsO) is a common chronic inflammatory skin disease affecting 1–3% of the general population (1). Approximately 1–3 in 10 patients with PsO will eventually progress to psoriatic arthritis (PsA), a chronic progressive seronegative spondyloarthropathy, characterized by arthritis, dactylitis, and enthesitis, with a debilitating course if untreated (2). Overall, an estimated 80% of PsA cases occur after a pre-existing diagnosis of PsO (3).

In the last 30 years, increasingly effective treatments for PsA have been developed and successfully implemented, with overwhelmingly positive effects on prognosis and long-term disability. This has shifted the focus of attention from treatment to *treating*, that is, to the early implementation and monitoring of available therapeutics.

Early diagnosis of PsA is fundamental, since a diagnostic delay as little as 6 months is associated with a poorer response to treatment, whereas early intervention with immune-modulating or anti-inflammatory drugs is linked with improved clinical and radiographic outcomes (4). However, factors responsible for the evolution from cutaneous to synovio-entheseal inflammation in these patients are still widely unknown. An earlier PsA diagnosis would require a better definition of disease phases and an in-depth characterization of the genetic, environmental, and immune-related events that precede PsA. In order to do so, large quantity of data must be generated through continuous access to rheumatological services and close clinical monitoring.

At present, the development of predictive models for PsA is impeded by a number of obstacles at multiple levels: at the macro level of diagnosis (i), at the meso level of access to specialist rheumatological cures (ii), and at the micro level of symptoms and disease monitoring (iii).

(i) Diagnostic delay currently represents one of the major challenges in rheumatology. The transition from PsO to PsA may either go unnoticed or be acknowledged with a major delay, leading to a poor window of treatment opportunity as well as to significant data loss (5–8). As musculoskeletal symptoms such as arthralgias, myalgias, and asthenia are quite common in the general population, it may be hard for general practitioners to correctly identify rheumatic symptoms that are indicative of an emerging PsA at its early stages (9). Also, patients may overlook their own musculoskeletal symptoms and wait for a spontaneous resolution or treat them with self-care methods (10).
(ii) Early access to rheumatological care is made difficult by a heavy workforce gap. Despite the global burden of chronic musculoskeletal disease being steadily on the rise, rheumatologist remain scarce and the global need of rheumatological cures cannot be met (11–13). The lack of sufficient numbers of rheumatologists has left some communities underserved and aggravated the problems of waiting times and diagnostic delay.
(iii) Even when a diagnosis of PsA has been suspected or ascertained, effectively monitoring the course of a rheumatic disease can prove to be a difficult task. While more frequent rheumatological visits are associated with greater improvements in pain and functionality (14), a sufficiently close monitoring of disease activity and patient-reported parameters is often not possible due to the relapsing-remitting nature of rheumatic diseases itself. Even in the occurrence of an exacerbation, an immediate rheumatological assessment is rarely a possibility and patients usually see their rheumatologist only after the disappearance of symptoms. Lastly, the advent of the coronavirus disease 2019 (COVID-19) pandemic further aggravated the problem, as many consultations had to be canceled or postponed (15–17).

Within this framework, the implementation of eHealth may step in to fill the gap. e-health interventions may offer a significant opportunity to both clinician and patients, as they promise to simultaneously improve disease monitoring and support self-management. e-health is already being extensively used beyond rheumatology and, to a lesser extent, in the management of rheumatoid arthritis. Thus, the question arises whether a similar change of paradigm in the current MoC for PsA could meet new necessities of patients and clinicians.

In this review, we cover current evidence on digital approaches in the early diagnosis of PsA in the PsO-PsA transition. Moreover, we review current eHealth tools for arthritis and discuss their possible implementations in this field.

## e-Health Approaches for the Management of Psoriatic Arthritis

**Figure 1:**
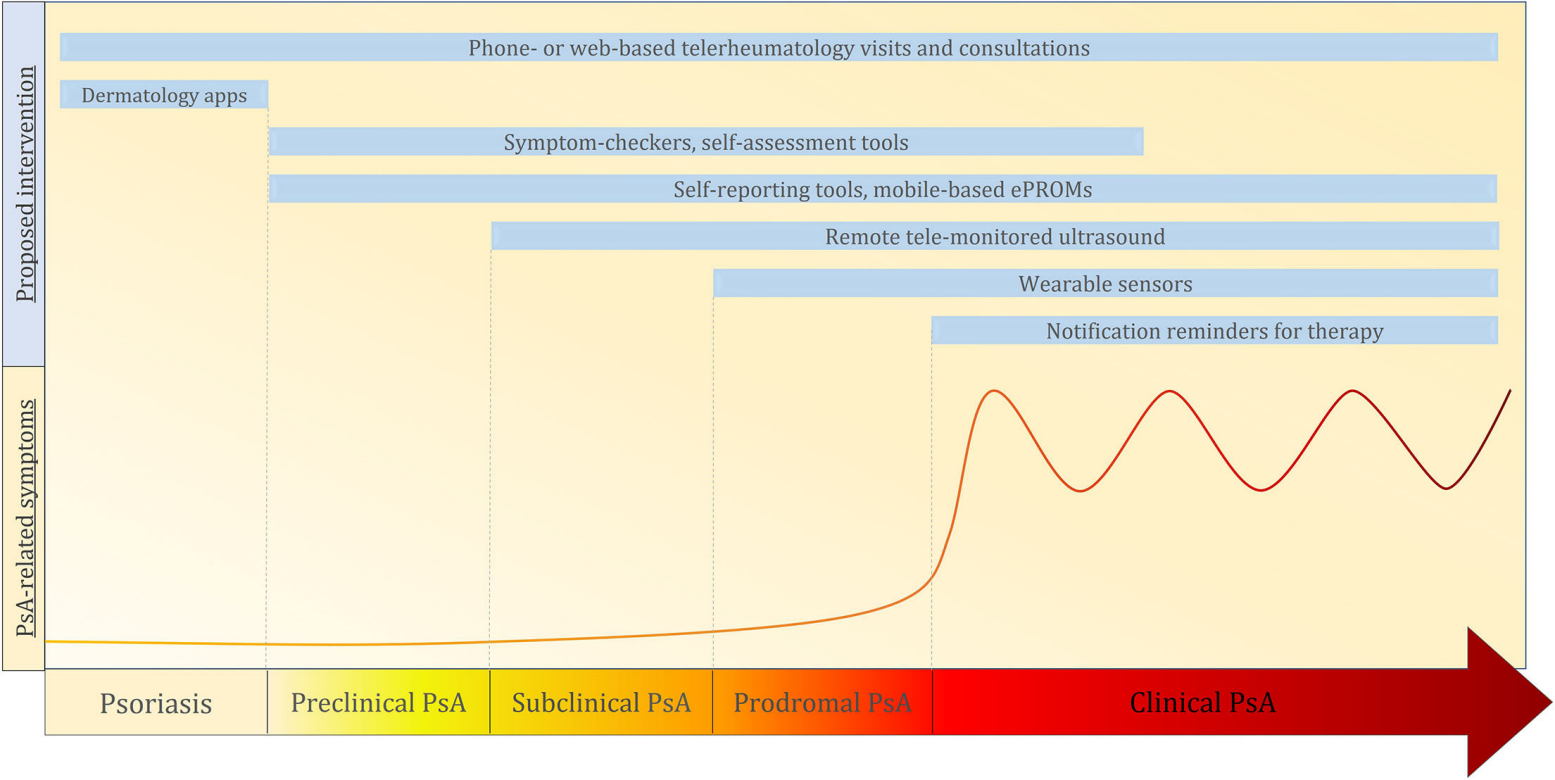
The arrow with the yellow-red gradient represent the progression phases from psoriasis to psoriatic arthritis (from left to right). In genetically predisposed patients with psoriasis, interactions between genetic end environmental factors may lead to disease progression toward arthritis. In the preclinical phase, no clinical, serological, or imaging alterations are detectable. It is proposed that this phase is characterized by the beginning of an aberrant innate immune response in key tissues (i.e., skin, entheses, intestinal mucosa). The following subclinical phase is characterized by the presence of serum biomarkers and imaging alterations of musculoskeletal change without clinically symptomatic arthritis. This is followed by a phase of prodromal PsA in which fatigue and non-specific musculoskeletal symptoms may occur. The last stage is that of a fully blown clinically symptomatic Psoriatic Arthritis. The curve represents the burden and frequency of arthritis-related symptoms during each phase. The light blue boxes indicate the possible eHealth interventions for the management of the Psoriasis/Psoriatic arthritis progression. PsO, Psoriasis; PsA, Psoriatic Arthritis; ePROMs, electronic patient-reported outcome measures.

e-Health is defined by the World Health Organization (WHO) as a collective term comprising the use of digital, mobile and wireless technologies to achieve health-related information, resources, and services (18). Examples of e-health include electronic medical records, telemedicine, symptom checkers, and mobile health (mHealth). As modern issues in the field of PsA management span across all levels of healthcare systems, the possibility of a “one-size-fits-all” solution appears highly unlikely. e-health can provide a diversified set of digital tools that may offer both parties—the patient and the clinician—new opportunities of information and knowledge exchange, as well as new more effective methods of disease monitoring. A number of digital approaches have already been tested both inside and outside rheumatology, and may bear great potential for application in the different stages of PsA (Figure 1).

### Telemedicine

Telemedicine is the practice of medicine at a distance using an electronic mean of communication (19). All interventions, diagnoses, and therapeutic recommendations are based on patient information and data that are either synchronously or asynchronously transmitted through telecommunication systems (i.e., by telephone, e-mail, video conferencing). The use of telemedicine in rheumatology (telerheumatology) can facilitate the dematerialization of several processes that are normally hampered by deficient infrastructures or personnel (e.g., renewal of medical prescriptions, follow-up visits for long-term stable disease) (20, 21). This could effectively improve the accessibility to rheumatological care and help bridge the workforce gap by increasing the capacity of referral centers (22). The effectiveness of telerheumatology could be equal to or greater than face-to-face outpatient clinic visits in terms of diagnostic accuracy and remission induction, especially in patients with low disease activity or remission (23–27).

From a patient's point of view, a very positive attitude toward telemedicine in general and its use in rheumatology has been reported in multiple studies (23, 28–30). Most notably, in a German survey study on rheumatological patients 64.4% of participants declared they would rather use telemedicine instead of regular in-person visits during follow-up (31). Surveys have also shown that general practitioners, and to an even greater extent rheumatologists, would welcome the addition of telemedicine to routine care (31, 32). This, however, is in sharp contrast with the actual numbers of telemedicine use in rheumatology (31). A survey from the American Medical Association showed that short of 10% of U.S. rheumatologists used telemedicine, a relatively low number and significantly less than other specialists such as radiologists, which attested at around 43% (33). Other surveys also found similar results, suggesting the existence of significant difficulties in implementing telerheumatolgy at a global level (31, 34). Organizational matters, rather than clinical ones, were described as the main barriers to the introduction of telemedicine by physicians. These include limited knowledge of the topic and the need for specific training, excessive administrative expenses for the purchase of equipment, and lacking reimbursement opportunities (31).

However, with the sudden begin of the COVID-19 pandemic, an unprecedented resort to telemedicine took place worldwide. Drastic changes in the organization of rheumatological care had to be made in little time due to infection control policies aimed at reducing in-person visits (35, 36). This would have meant canceling or postponing large numbers of appointments, leaving patients unable to receive adequate rheumatological treatment. A common solution to this critical scenario came from the application of basic telemedicine, that is, by converting in-person visits to telephone or video consultations whenever possible (15, 37, 38). In the case of patients suffering from PsO, PsA, and other kinds of inflammatory arthritis, this approach has been well-documented in the literature and has involved large numbers of participants (39–41). As a result of the pandemic, it seems that patient and physician disposition toward telemedicine has improved, as well as the willingness to promote such instruments by health institutions (29, 32). This unusual opportunity is likely to accelerate the use of telemedicine and foster the development of new healthcare standards (42).

Telerheumatology also comprises several instruments that can be exploited by both physicians and patients for diagnosing, monitoring, and self-monitoring. The use of wearable sensors has already been experimented in rheumatoid arthritis to passively monitor hand mobility, step count, and energy expenditure and could easily find implementation in PsA as well (43, 44). The body-worn sensors generate data that can accurately reflect disease activity, flareups, and the degree of functional disability after a therapeutic intervention (45). Results can then be visualized to therefore provide individual support and recommendations and to improve therapy management and outcomes (46). Imaging is also an important factor for the detection of disease activity. Ultrasound is a cost effective and highly sensitive technique (47). Remote tele-mentored ultrasound (tele-ultrasound) is a tool that allows the sonographic evaluation of patients from a distance in a synchronous or asynchronous manner (48). It utilizes a single centralized physician with training in ultrasound interpretations alongside a bedside operator that performs image acquisition under the guidance of the former. This technique has already demonstrated its feasibility and accuracy in critical care applications (49), and even on the international space station (50). So far in rheumatology no reports are available, but the potential of this technique could be a good match in the early diagnosis of PsA. It is already known that a relevant portion of patients with PsO will exhibit subclinical joint and entheseal inflammation/disease before developing a clinically patent form of arthritis (51–53). Many of these patients, however, have no access to sonographic examination at their general practitioner's or in the dermatology clinic. Implementing tele-ultrasound of key articular and entheseal structures under the guidance of a rheumatologist may prove as an accurate and cost-effective solution. This may greatly contribute to the early diagnosis of PsA while simultaneously bypassing the problem of low accessibility to rheumatologists.

Evidence in support of an unrestricted use of telerheumatology, however, is currently limited. From a critical point of view, the loss of physical contact and the difficulties that emerge in establishing an emotional relationship between patient and physician have been regarded as the drawbacks of telemedicine (54, 55). In addition, problems of social nature may also emerge, as elderly and socioeconomically disadvantaged patients may lack the necessary skills or equipment to use telemedicine (56). In general, a lack of sufficient eHealth literacy has been found in both patients and physicians and needs to be addressed by offering specific education (34, 57). Further studies are needed to determine the best applications and conditions for the use of telemedicine, and especially a more rigorous assessment with specifically designed randomized clinical trials (58). Also, many of the available tools yet lack validation for PsA. It is likely that in the future patient- and physician-adapted telemedicine options will emerge, with the possibility of triaging patients for either digital or in-person consultations, as more appropriate for the specific situation (59, 60).

### mHealth

Modern treatment options for PsO and PsA include both pharmaceutical and non-pharmaceutical interventions. They require long-term adherence to medication regimen, phototherapy and physical therapy sessions, as well as a regular follow-up schedule. This increase in the complexity of the management of psoriasis has left many patients unwilling or unable to closely adhere to treatment plans (61). Tangible help in self-management and personalisation may come from mobile technology. The WHO defines mHealth as “*the use of mobile and wireless technologies to support the achievement of health objectives”* (62). Smartphone and mobile devices ownership is growing rapidly around the world. In Europe and the United States, over three quarters of the adult population are active smartphone users and use internet-based applications daily (63). The use of mobile devices for health purposes has thus become an increasingly accessible commodity for many, and numerous health-related smartphone applications are now available (64, 65). These include symptom checkers (66, 67) and referral tools (68) that could be helpful for the screening of early arthritic manifestations in PsO, as well as applications for the passive and active monitoring of disease activity [e.g., wearable sensors applications for the passive monitoring of key disease features (45, 69, 70), self-reporting tools (71)]. Medication adherence could also be improved via notification reminders on personal smartphones (72–74).

mHealth holds great potential for getting patients with rheumatic diseases involved in the self-management of their conditions, as well as for improving communications with their health care providers (75). Rheumatic patients already showed a propensity toward the use of mobile technologies to track their symptoms and disease activity in various surveys, and declared themselves optimistic about the utility of mHealth (32, 57). Sharing disease-related data for research purposes was also regarded favorably (32). Furthermore, rheumatologists themselves have increased their use of mobile health applications in recent years (33, 76). As with telehealth, COVID-19 acted as a catalyser for the acceptance of mHealth for both patients and physicians (32).

From a rheumatologist's perspective, mHealth can allow real-time symptom and disease activity monitoring and rapidly generate large quantities of patient-reported measures for clinical and research purposes (57, 77–79). Electronic patient-reported outcome measures (ePROMs) have many advantages compared to paper-based alternatives. Firstly, they seem to be preferred to traditional paper forms (80–82) and lead to comparable results (83). Furthermore, data collection through ePROMs is easier and faster, and completely bypasses the step of data digitalization (81). This could make the inclusion of PROMs into clinical practice more feasible for many rheumatologists. Indeed, a main reason for not implementing ePROMs is the unawareness of suitable software solutions (84). Also, sharing PROM results with patients can improve their knowledge of the disease and improve adherence by consolidating trust (85).

In the case of PsO, Schreier and colleagues developed a mobile phone-based teledermatology app for the real-time reporting of active psoriatic lesions through photographs (86). Similar applications, if integrated with further functionalities such as symptom checkers and monitoring tools, may facilitate the identification of patients at higher risk of developing arthritis and improve treatment outcomes for those who were already diagnosed.

Generalized enthusiasm toward mHealth, however, is not always matched by sufficient levels of competence with mobile technology. Studies found poor levels of literacy in this field, especially in older patients (57). Furthermore, analysis of the available applications for common smartphone operating systems found a lack of high-quality apps in terms of scientific accuracy and compliance to evidence-based guidelines (87–89). To address and reduce these limitations, the European alliance of associations for rheumatology (EULAR) has recently published recommendations for the development of mobile rheumatology applications (90). Nonetheless, specific studies demonstrating the effects of mobile applications in the field of rheumatology are direly needed.

### Symptom Checkers and Combined Tools

Symptom checkers are artificial-intelligence (AI)-based tools designed to help patients determining the possible causes of their symptoms and eventually direct them to medical attention. In recent years, the use of internet- and mobile- based support for self-diagnosing has greatly increased, and an increasing number of patients perceive symptom checkers as useful for diagnosis (67, 91). In rheumatology, symptom checkers could help reducing diagnostic delay by ensuring quicker referral of patients who are at higher risk of having or developing a rheumatic condition. Bechterew-check is an online patient-facing self-referral tool for patients with chronic low back pain and has been developed for the early recognition of axial spondyloarthritis (92). This tool proved effective in the detection of previously undiagnosed cases of spondyloartrhitis by bringing high-risk patients to medical attention on the basis of clinical and demographic parameters. While holding great potential for early diagnosis, rheumatologists are not yet keen to recommend symptom checkers, as reliability remains one of the major drawbacks and there is still little evidence in their support (93). The full potential of symptom checkers, therefore, may only be reached in combination with other objective disease measures (94).

The Joint Pain Assessment Scoring Tool (JPAST) is an European Union-funded project on digital health in rheumatology that aims at combining eHealth with the analysis of validated biomarkers (95). The JPAST acts as a digital prognostic program by merging patient symptom checker inputs with serological and genetic data. This approach could greatly improve the early identification of patients at high risk of developing rheumatic diseases as well-facilitating a timely intervention.

Since risk stratification is the main conundrum of the PsO-PsA transition, combined eHealth tools such as JPAST hold great potential in this field. The generation and analysis of high-quality data is likely to allow to pre-emptively identify PsA patients in their pre-clinical phases, and to adjust therapeutic interventions and monitoring accordingly. Patient-centered eHealth platforms for spondyloarthritis such as SpA-NET in the Netherlands (96) and SpAMS in China (97) have already been developed and successfully implemented in clinical practice. This highlights the possibilities for eHealth in PsA management. In the future, the full potential of combined tools in rheumatological practice should be explored in more specific studies.

## Digital Rheumatology in Psoriatic Arthritis Research

As research questions in rheumatology become more complex, finding the right answers requires growing quantities of patients and data that cannot always be provided by traditional clinical research. Electronic health has the potential to bridge this gap by using digital tools to increase data output while simultaneously reducing administrative efforts (57). With the possibility of rapidly generating large high-quality datasets, it is likely that innovative data collection systems such as digital crowdsourcing will be key elements for answering future research questions. Digital crowdsourcing is a technique of data collection that gathers the collective outputs of large numbers of participants (i.e., patients, clinicians) through technological means to rapidly provide answers to specific questions (98). Continuously generated data may be further elaborated and analyzed by AI and used as a basis for machine learning systems, that in turn can be trained to detect disease activity and flareups (99), predict therapy responses (100), and patient phenotyping (101, 102).

## Conclusions

The traditional authoritative MoCs in which physician were the sole arbitrators in all matters of patients' therapies revealed several limitations in managing the PsO-PsA transition. This is due to both the high complexity of the issue and to the absence of effective means of collecting relevant disease-related data with traditional methods. Modern MoCs for PsO and PsA emphasize the importance of patient engagement and self-management (103). Within this context, eHealth approaches such as telemedicine, mHealth, and combined tools offer important opportunities to complement rheumatology care in order to establish timely and more accurate diagnoses, and to intercept high-risk PsO patients before their clinical transition toward arthritis (Figure 1). Previous experiences indicate that screening for at-risk patients is feasible through the monitoring of clinical and biometric parameters (e.g., pain, body weight, physical activity) (2), early imaging (51, 53, 104), and through screening questionnaires (105). At the moment, however, there are too little data to establish a definite strategy for the integration of eHealth in this aspect of PsA management. Until no validated tools for PsA are developed and tested in clinical trials, the potential of eHealth in this field is therefore bound to remain partly speculative. Digital solutions have already been implemented with promising results in rheumatoid arthritis (106, 107), and have the potential to be transferred and adapted to PsA. If successful, these measures could address diagnostic delay and low treatment adherence, improving disease monitoring and eventually leading to better disease outcomes.

## Author Contributions

FF and DS: literature research and manuscript drafting. FF, JK, AK, and DS: conceptualization. KT has expertise in the field of digital rheumatology and critically revised the manuscript. He also contributed to the revision phase. All authors contributed to the article and approved the submitted version.

## Conflict of Interest

The authors declare that the research was conducted in the absence of any commercial or financial relationships that could be construed as a potential conflict of interest.

## Publisher's Note

All claims expressed in this article are solely those of the authors and do not necessarily represent those of their affiliated organizations, or those of the publisher, the editors and the reviewers. Any product that may be evaluated in this article, or claim that may be made by its manufacturer, is not guaranteed or endorsed by the publisher.
